# How can we Challenge the Four-minute Rule?

**DOI:** 10.1016/j.ebiom.2016.03.029

**Published:** 2016-03-24

**Authors:** Katsuhiko Naruse

**Affiliations:** Department of Obstetrics and Gynecology, Nara Medical University, 840, Shijo-Cho, Kashihara 6348521, Nara, Japan

A manuscript entitled “Maternal Collapse: Challenging the Four-minute Rule” by Benson et al. (2016)), published in *EBioMedicine*, proposed a new point of view for perimortem Cesarean section (PMCS): one procedure intending not only to save the fetus, but for resuscitation of pregnant women after cardiopulmonary arrest after 20 weeks of gestational age. In obstetrics, obstetric anesthesia, and emergency medicine, the “Four-minute Rule” is regarded as a gold standard for the decision of PMCS; maternal recovery rate after PMCS was significantly decreased when the fetus was delivered over 5 min after maternal cardiac arrest, and a “skilled” obstetrician was expected to complete Cesarean delivery within 1 min of incision. (American Society of Anesthesiologists Task Force on Obstetric Anesthesia, 2007) However, Benson et al. posed the question on this standard that the injury-free survival for mother and baby showed no unique difference between four and five minutes (or any other minutes) of arrest to birth time. And as was expected, their search of the literature proved that many reports of PMCS showed durations longer than 1 min between incision and delivery, regardless of skill.

One manuscript describing PMCS from the trainer's point of view was published in 2010 (Dijkman et al., 2010) and showed the increased number of PMCS after training of the procedure. However, in this report, no case of PMCS was completed within 5 min (although 2 out of 3 mothers and all fetuses survived after PMCS 5 to 15 min after arrest), similar to another report to which Benson et al. referred (Einav et al., 2012) (only 4 in 57 cases of PMCS performed and return of spontaneous circulation achieved). The most significant reason for delayed delivery may be hesitation to carry out PMCS; Dijkman claimed that there were no survivors of “out-of-hospital” arrest, even if PMCS was performed (Dijkman et al., 2010).

What, then, can we propose next to universalize and improve the results of PMCS? Below are my personal recommendations, beyond the manuscript by Benson et al.: [1] making decisions more quickly, just as quick as possible, [2] changing the 4-minute rule to longer minutes like 10, 15 or 25 min, [3] training all obstetricians or emergency room doctors to complete PMCS within 1 min, or [4] beginning immediate use of mechanical (possibly percutaneous) cardiopulmonary support (or the invention of a new machine usable after shorter preparation). Since [3] and [4] are not realistic, [2] may be the easiest and most reasonable, given that former reports showed expectations of maternal survival at more than 5 min but less than 10 or 15 min (Dijkman et al., 2010, Einav et al., 2012), but as a “rule”, it seems too long. The option [1], proposed in this manuscript by Benson et al., is another possible tactic, but it may cause harsh mental pressure on bystanders to make a decision of non-anesthetic cesarean section within 2 or 3 min after arrest, as it allows only one cycle of cardiac massage and automated external defibrillator (AED) use on basic life support (BLS). However, the latest recommendation by the International Liaison Committee on Resuscitation (ILCOR) also specified that there was no specific time interval by which delivery should begin, which Benson et al. clearly supported in this manuscript (Soar et al., 2015). At least, we must modify the process flow to include PMCS from when we initially encounter cardiac arrest in pregnant women, as a new recommendation.

## Disclosure

The author declared no conflict of interests.
